# Some Miscellaneous Reflections on Dentistry and Dentists

**Published:** 1882-10

**Authors:** F. W. Sage


### Some Miscellaneous Reflections on Dentistry and Dentists.

F. W. SAGE, D.D.S.

The history of the rise, development and decline of trades, furnishes suggestions and warnings which are not without significance to those who are engaged in what has been called the *semi-professional* occupation of dentistry. This appellation we take it, is applied in consideration of the combined surgical and mechanical nature of the profession; not necessarily in a derogatory sense. Accepting the term for what it appears truly to indicate, and regarding the subject, for once, from a *selfish* stand point, if the reader pleases, we come to consider, what are the influences, which, operating at the present time, seem to threaten the members of our profession with loss, both as regards the dignity of their vocation and adequate remuneration for services performed. The writer takes the liberty, *en passant*, of saying that he is not quite in sympathy with a sentiment which frequently finds expressions in our conventions, to the effect that the question of compensation is not worthy of equal consideration with the question of achieving the most desirable results for our patients. To assume that, is practically to assume that a large majority of the members of the profession are engaged in missionary work, for it is beyond all question true that most of us find the supply greatly in excess of the demand, as regards opportunities for gratuitous service. If the object desired to be accomplished by this inculcation be to sustain a high standard of attainment in dentistry by stimulating the members of the profession to exercise their best ability, we question whether one important means to this end (to wit, the consideration of the dentist's duty *to himself*) has been sufficiently regarded. Taking human nature as we find it (among all classes of people, dentists included,) we suspect that lack of appreciation, manifest ingratitude, and a niggardly spirit on the part of patients, have frequently so operated to discourage and demoralize the dentist, as to chill enthusiasm, relax ambitious resolve, and induce bitter repinings. How would this do by way of varying the old injunction? "Use your very best efforts for those who are able to pay you and, in justice to yourself, be exceedingly chary of underestimating the value of your service by accepting anything less than your regular fee." If this be heresy make the most of it.

Is it wise or just to encourage young men to surround themselves by a class of mendicants who possibly are not entitled to the *luxury* of first-class dental operations? Let us put aside all nonsense in this matter and press the claims of our enlightened profession for a while. If our impecunious patient cannot afford a Richmond crown, by what principle of justice are we required to make him a present of one? Or if recognizing our claim to adequate compensation he modestly expresses a preference for the cheaper plate, shall we refuse him the request because it does not accord with our views of what would be the very best thing to do? Yet we are constantly reminded that it is malpractice to extract a root which might be saved. Let us consider *that* matter a little.

The ethics of all professions may be regarded as resting upon the principle that there is no middle ground in practice between good and bad. The lawyer who practices conscientiously must use as much care in drafting a deed as other legal instrument, for the person who pays little as for the person who pays much. It is not sufficient that he *half* acquits the client who *half* pays him; he must do his whole duty and wholly acquit him if possible. The physician must *heal*, not *half* heal the patient he treats, whether he receives a full fee or only part. The surgeon must amputate the pauper's limb as carefully as he would the capitalists; the instincts of humanity cry shame on the surgeon who refuses his services until the fee is produced. But the surgeon's ally whose office it is to supply an artificial limb, consults the applicant's means. No appeal to his compassion and sympathy would be regarded as making it in the least obligatory upon him to furnish an expensive cork leg for the price of a wooden stump. It is nothing to him, from a business point of view, that the applicant's future comfort and convenience depend materially upon the style of artificial limb with which he is furnished. In short, it appears that the lawyer, the physician and the surgeon must perform services which look to the accomplishment of one definite, unvarying result. Anything short of this subjects the professional man to the imputation of malpractice. There is no middle course of action open to him. Is this equally true of the dentist? We think not, if only for the reason that the consequences attending a deviation from what would be regarded by the best judges of dental operations as the best thing to do, would hardly be regarded as affecting the patient's welfare in the same degree. We conceive that in all ordinary cases the dentist has discharged his whole duty when he has explained to his patient the advantages and disadvantages of different courses of procedure proposed. After that the responsibility should rest upon the patient, not upon the dentist. It is certainly no reproach to the dentist that he cannot always bring his patient to appreciate the value of his suggestions; whether or not this should be considered as justifying a refusal to operate for such patients, is a question for others to decide.

In discussing this question we have touched upon one of the evils which threatens the profession, especially as regards those who are struggling to get into practice. Expressed in other words, there is not that unity of spirit and purpose as regards the requiring of adequate compensation for valuable services, which is required to save the dignity and preserve the advancing standard of the profession. All that we have said is certainly not designed to encourage a mercenary spirit; it is rather designed to encourage ambition and to foster invention and ingenuity by placing wholesome restrictions upon those who would reap the benefits of our acquirements without adequate remuneration. The *laborer* is worthy of his hire, and how much the more so if the labor he performs is of such a nature that only years of study and practice will enable him to achieve successful results? We opine that to preserve a high standard of excellence and to sustain at the same time a proper respect for self and for ones calling, in the face of such discouragement as comes from poorly requited services, is more than can be expected of most dentists, in the present imperfect state of human nature. We look all about us and what do we see? Dentists leading a bread-and-butter existence, toiling at a nerve-destroying occupation from morning till night, from youth till old age, and after all, seldom acquiring a competency. A lawyer of twenty-five years' experience recently remarked in the writer's hearing, that he could now try a case with one-fourth part of the labor that was required during the earlier years of his practice. The physician's cares and difficulties, if not his responsibilities, grow lighter with the increase of experience. It is not the case, in anything like the same degree, at least, with the dentist. He rests under the disadvantage of labor ever increasing while his natural powers are ever declining All of which argues that the dentist, of all professional men, should reap his harvest as early in life as possible, if he is mindful of the duty he owes himself and family.

Elevating the standard of dentistry is to be accomplished in no inconsiderable degree, by elevating fees. We claim originality, not for the discovery but for the promulgation of this doctrine. Let us require some of our flat-pursed patrons to divert their efforts in some other direction, when the question of curtailing expenses comes up for consideration. We question whether there can be found among any other class of professional men so many who hold themselves in a constant attitude of apology towards their patrons, when the matter of compensation is touched upon, as do dentists. There are many who need to remind themselves constantly that they are entitled to something more than mechanics' wages, in consideration of the arduous nature of their duties, the waste of nervous force, and the almost constant disadvantage under which they rest, of working under protest. Whenever a patient says to me, "I wouldn't be a dentist for the world," I usually improve the opportunity to impress him profoundly with the conviction that it requires considerable of an outlay to hire *me* to be a dentist. But what of the future of dentistry, since, of late years so many young men are entering the ranks of the profession? Is it possible that the time will come when it will be necessary to form combinations like the trades unions to protect ourselves in the pursuit of a livelihood? Or may we reasonably expect that with the dissemination of knowledge the large majority, who at this day seek no service at our hands, will swell the ranks of patrons so as to preserve the equilibrium? The latter supposition would seem the more reasonable of the two, but it must be considered that in all probability most of those who do not avail themselves of our services at this day are deterred by pecuniary considerations. That in turn would seem to indicate that with the increase of the number of dentists prices must decline. One circumstance which may be expected to offset this threatened disadvantage is the prospect that with the increase of the ravages of decay of teeth will come an increase of the demand for professional services on the part of the present classes of our patrons. It is hardly to be expected, despite the efforts of scientists to discover and correct hygienic errors and abuses, that the *greater* calamities which threaten the teeth of coming generations will be checked to any appreciable extent so long as civilization continues to develop and nourish luxurious and enervating habits in the commonwealth. There will undoubtedly then be ample room and sufficient demand for all the first-class men the colleges can supply for many years to come; so let the college boom go on, we say.

The everlasting amalgam question next. The decision of that question, for or against, is certain to affect practice very materially. If the result be to encourage its use there will be a still farther decline in the demand for gold in back teeth. The effect of this will be to let down the bars and admit charlatanry into the domain of operative as well as mechanical dentistry in all probability. Not, however, to the same extent, by any means, for the reason that gold will still be used in front teeth, and here the failures of incompetent men will serve to advertise more conspicuously their general incompetency. At all events if the use of amalgam could and should receive the sanction of competent investigators, the encroachment of amalgam upon the present domain of gold will be undoubtedly very gradual. We are not to be understood as making any predictions favorable or unfavorable to the use of amalgam.

If, on the other hand, this material be unanimously condemned by the same authorities some of us might find ourselves perplexed in attempting at once to discard its use. How, for instance, would Dr. A., who is consulted by Miss B., with reference to an inexpensive filling for her back teeth, explain without embarrassment or complication (in the presence of Miss B.'s sister, for whom he inserted amalgam fillings a few months before) that amalgam had been pronounced highly injurious? Well, Dr. A. would need to exercise a certain degree of ingenuity. We cannot say exactly *what* Dr. A. would need to tell her. If Dr. A. were a church member he would probably need to keep that fact steadily in view, if his object was to preserve his veracity and at the same time avoid awakening any uneasiness on the part of Miss B. No. 2. The alternative would probably be to remove the amalgam fillings from the mouth of Miss B.'s sister and insert some kind of cement, *gratis*, or for all above that which his self-possession would permit of his charging. One consolation that Dr. A. might hug to his bosom would be derived from the reflection that but few of his former patrons, in whose mouths were amalgam fillings, would be likely to take the alarm long before symptoms of dissolution set in. People are not so fond of having amalgam bored out, as to resort to that expedient until thoroughly convinced that evil is being wrought by its presence in their mouths. And this brings us to the reflection that perhaps after all, the decision of this amalgam question rests more with the public than with us of the profession, if the truth were known.

